# Ultra-processed food consumption is associated with increase in fat mass and decrease in lean mass in Brazilian women: A cohort study

**DOI:** 10.3389/fnut.2022.1006018

**Published:** 2022-10-14

**Authors:** Lívia Carolina Sobrinho Rudakoff, Elma Izze da Silva Magalhães, Poliana Cristina de Almeida Fonseca Viola, Bianca Rodrigues de Oliveira, Carla Cristine Nascimento da Silva Coelho, Maylla Luanna Barbosa Martins Bragança, Soraia Pinheiro Machado Arruda, Viviane Cunha Cardoso, Heloisa Bettiol, Marco Antonio Barbieri, Renata Bertazzi Levy, Antônio Augusto Moura da Silva

**Affiliations:** ^1^Post-Graduate Program in Collective Health, Public Health Department, Federal University of Maranhão, São Luís, Brazil; ^2^Nutrition Department, Federal University of Piauí, Teresina, Brazil; ^3^Post-Graduate Program in Collective Health, State University of Ceará, Fortaleza, Brazil; ^4^Post-Graduate Program in Child and Adolescent Health, University of São Paulo, Ribeirão Preto, Brazil; ^5^Department of Preventive Medicine, School of Medicine, University of São Paulo, São Paulo, Brazil

**Keywords:** ultra-processed foods (UPFs), food consumption, diet, body composition, body fat

## Abstract

**Objective:**

To investigate the association between ultra-processed food consumption at 23–25 years of age and measurements of body composition–fat mass, fat mass distribution and lean mass at 37–39 years of age in Brazilian adults.

**Methods:**

1978/1979 birth cohort study conducted with healthy adults from Ribeirão Preto, São Paulo, Brazil. A total of 1,021 individuals participated in the fat mass analysis (measured by air displacement plethysmography) and 815 in the lean mass analysis and fat mass distribution (assessed by dual energy X-ray absorptiometry). Food consumption was evaluated by a food frequency questionnaire. Food items were grouped according to the level of processing as per the NOVA classification. Ultra-processed food consumption was expressed as a percentage of total daily intake (g/day). Linear regression models were used to estimate the effect of ultra-processed food consumption (g/day) on body mass index, body fat percentage, fat mass index, android fat, gynoid fat, android-gynoid fat ratio, lean mass percentage, lean mass index and appendicular lean mass index. Marginal plots were produced to visualize interactions.

**Results:**

The mean daily ultra-processed food consumption in grams was 35.8% (813.3 g). There was an association between ultra-processed food consumption and increase in body mass index, body fat percentage, fat mass index, android fat and gynoid fat and decrease in lean mass percentage, only in women.

**Conclusion:**

A high ultra-processed food consumption is associated with a long-term increase in fat mass and a decrease in lean mass in adult women.

## Introduction

The body composition assessment is important to describe and monitor nutritional status with accuracy, in addition to several physiological processes and pathological conditions, making it possible to identify individuals at risk and plan appropriate therapeutic interventions (1, 2). Excess fat mass (FM) is related to higher morbidity and mortality, and the metabolic risk related to fat accumulation is strongly dependent on its distribution (3). The monitoring of lean mass (LM) allows the identification of cachexia, sarcopenia and sarcopenic obesity, situations that are related to increased functional decline and high risk of disease and mortality (2).

Several factors affect body composition, and diet and level of physical activity are some of the most important aspects to be considered (4, 5). Low-carbohydrate diets, for example, may be effective in decreasing FM in obese individuals (6), whereas high-protein diets may have a beneficial effect on weight loss, increasing FM loss and preserving fat-free mass (FFM) (7). However, the diet effect on body composition varies according to age, nutritional quality of this diet, nutritional status, sex of individuals, level of physical activity, hormonal load, genetics, and lifestyle, among other factors (8–10).

The nutritional quality of diets has been widely evaluated using the NOVA food classification system, which groups food according to the nature, purpose and extent of industrial processing rather than in terms of nutrients and food types (11–13). Food items are grouped into 4 groups: Group 1 - Raw or minimally processed foods; Group 2 - Processed culinary ingredients; Group 3 - Processed foods, and Group 4 - Ultra-processed foods (UPF) (11–13), the last consisting of industrial formulations made from numerous food-derived ingredients, with little or no whole food, often added with additives that modify their sensory attributes. UPF has, on average, higher energy density, more free sugar, more total and saturated fats and lower concentrations of fiber, proteins, vitamins, and minerals (11–13). Thus, diets with high UPF participation have their nutritional quality impaired (14–18).

UPF is associated with increased overweight, abdominal obesity, hypertension, coronary and cerebrovascular diseases, dyslipidemia, metabolic syndrome, gastrointestinal disorders, diabetes mellitus, depression, asthma, frailty in the older people, total and breast cancer, and increased overall mortality (14–18). In Brazil, according to the 2017-2018 Household Budget Survey (POF 2017–2018), the mean UPF contribution to the total dietary energy intake (TDEI) of the diet was 19.7% (19).

Although an association between UPF consumption and excess weight was demonstrated (20–24), few studies estimated its long-term effect on body composition. Costa et al. (25) found that a 100 g increase in the UPF contribution to the daily intake of children at age six was associated with a gain of 0.14 kg/m^2^ in the fat mass index (FMI) at age 11. In adolescents, a 1% increase in the UPF contribution percentage to total dietary energy intake was associated with a 0.04 kg decrease in muscle mass (MM) and a 0.01 kg/m^2^ decrease in lean mass index (LMI) (26). A cohort study that evaluated associations between UPF consumption and adiposity trajectories from childhood to early adulthood (from seven to 24 years of age) showed an annual increase of 0.03 kg/m^2^ in FMI in the fifth quintile of UPF consumption when compared to the first (27).

To date, to our knowledge, only one longitudinal study evaluated the association between high UPF consumption and changes in body composition (assessed by great validity methods) in adults and older people. Individuals aged 55–75 years with metabolic syndrome, overweight and obesity were followed for 12 months and it was found that a 10% daily increase in UPF consumption was associated with an accumulation of 0.09 g in visceral fat, 0.05 in android-gynoid fat ratio (AGR), and 0.09% in body fat percentage (BFP) (28). No studies were found that evaluated the UPF effect on the LM of adults.

In this sense, the aim of this study was to investigate, prospectively, the association between UPF consumption at 23–25 years of age and the composition and distribution of body fat and lean mass at 37–39 years in adults followed by the 1978/1979 Ribeirão Preto birth cohort study (São Paulo, Brazil).

## Methods

### Study design and study population

This is a prospective cohort study with data from a matrix research entitled “Estudo epidemiológico-social da saúde perinatal em Ribeirão Preto, SP” (29). Data from three of the five phases of the cohort study were used: baseline (birth) (1978/1979); the fourth phase, at 23–25 years of age (2002/2004), and the fifth phase, when the participants were between 37 and 39 years old (2016/2017). At birth (baseline), 6,827 children born in hospitals to mothers residing in Ribeirão Preto were evaluated. In the fourth phase, 2,063 individuals were evaluated, characterizing 30.2% of the original cohort. In the fifth phase, data were collected from 1,775 participants. Further details of each cohort stage, its objectives, methods, and eligible individuals were previously published (29, 30).

For this study, the assessment encompassed only those born in a single birth, who participated in the fourth and fifth phases of the cohort, whose food consumption data were collected at 23–25 years and body composition data were evaluated at 37–39 years, totaling 1,050 individuals for the analysis of fat mass (Body Mass Index–BMI, BFP, FMI) and 839 for the analysis of lean mass (percentage of muscle mass–LMP, LMI and appendicular lean mass index -ALMI) and distribution of body fat (android fat–AF, gynoid fat–GF and AGR). Individuals at the extremes of caloric intake (above or below three standard deviations of the mean total caloric intake, *n* = 08) and at the extremes of body composition (below the 1^st^ percentile and above the 99^th^ percentile) (21 for adiposity and 17 for lean mass and distribution of body fat) were excluded, totaling 1,021 individuals for analysis of adiposity and 815 for analysis of lean mass and distribution of body fat. In Supplementary Figure S1, a detailed flowchart of the cohort phases is shown.

### Food consumption assessment (exposure variable)

Food consumption was assessed using a semi-quantitative Food Frequency Questionnaire (FFQ) adapted for use in programs for the prevention of Chronic Noncommunicable Diseases (NCDs) aimed at adults, including the age group assessed in this study (31). The questionnaire was applied by nutritionists, with the aid of a photo album to help estimate the portions consumed. The FFQ contained 75 food items, referring to food consumption in the last 12 months, with options for the number of times (0–9) and the frequency of consumption (daily, weekly, or monthly), and the reference average portion size, for the individual to estimate whether the portion usually consumed was small (smaller than that presented), medium (equal to that presented), or large (greater than that presented) (32). The food portions were classified according to the percentage distribution of weights equivalent to the household measures referred to in the 24-h dietary recall (24 hDR), previously applied as part of the FFQ elaboration. The average portion presented in each food item represented the 50th percentile and the small and large portions, respectively, the 25th and 75th percentiles. The methodology of the validation has been described in detail by Molina et al. (32).

To obtain the consumption of each food item in grams (g) or milliliters (ml), the referred frequencies were converted into daily frequency and multiplied by the serving size. The nutrient and energy intake of each food item was calculated using food composition tables (33–35). In the case of alcoholic beverages, kilocalories from alcohol were also considered. The sum of calories from all food items reported in the FFQ made up the total calories consumed. The consumption of macronutrients, fiber and alcohol was adjusted to 1,000 kcal/gram.

Food items were grouped according to NOVA classification into raw or minimally processed, processed, and ultra-processed foods. Culinary ingredients were grouped together with the group of raw/minimally processed foods, in a group called “food preparations” (36). When food items from different groups were grouped in the FFQ, it was decided to divide the share of these food items into more than one group, by means of an estimate, using as a parameter the consumption observed in the state of São Paulo according to the Brazilian Household Budget Survey (2002–2003) (37), the closest to the data collection period of our presentation. Finally, the percentage of participation of calories and grams from UPF consumed by participants in the daily total of grams and calories, respectively, was calculated.

### Body composition assessment (outcome)

Body weight (kg) and height (cm) were evaluated in the fifth phase. Trained researchers performed these measurements using a high-precision scale coupled to an Air Displacement Plethysmography (ADP) device (COSMED BodPod^®^ Gold Standard, Concord, USA) with 100 gram graduation. Height was measured using portable AlturaExata^®^ stadiometers with 0.1 cm accuracy (Belo Horizonte, Brazil). Participants were assessed in an upright position, with the head oriented according to the Frankfort horizontal plane.

BMI was calculated by dividing weight in kilograms by height in square meters (kg/m^2^). Body adiposity was measured by LMI and BFP. This was evaluated by the ADP and converted into kg of fat mass, considering the individual's weight. The FMI was obtained by dividing the fat mass (kg) by the height in square meters (38).

To assess LM and body fat distribution, dual-energy R-ray absorptiometry (DXA) was used. The GE Healthcare^®^ Lunar Prodigy Densitometer (Chicago, United States) was used in a full-body scan with the participant immobile in the supine position on the table, with legs together and arms along the body. The body weight estimated by DXA was different from the weight obtained with the digital scale. Thus, it was necessary to adjust the MM by calculating the LMP estimated by DXA. The adjusted MM (kg) was then obtained by multiplying the LMP by the weight recorded on the digital scale (26). Appendicular lean mass (ALM) (kg) was calculated as the sum of the MM of the arms and legs. The MMI (kg/m^2^) was calculated as the ratio between the adjusted LM (kg) and the height in square meters, while the ALMI (kg/m^2^) was obtained as the ratio between the ALM (kg) and height in square meters (39).

Body fat distribution was assessed by measuring AF (kg) and GF (kg). The AF region comprised the space between the ribs and the pelvis, and the GF comprised the region relative to the hip and thighs. The lateral limits established were the lines of the arms when in a normal position for the full-body scan. Central fat distribution was measured through the ratio between AF and GF (AGR) (28).

### Other variables of interest

At 23–25 years of age, data were obtained by trained personnel using structured questionnaires, and the following variables were evaluated: sex (male/female); age categorized in years (23 years/24 years/25 years); race/ethnic group (white/black/brown/yellow or indigenous); family income in minimum wages (< 5 minimum wages/5–9.9 minimum wages/or ≥10 minimum wages); marital status (with a partner/without a partner); current smoking (yes/no); television (TV) and reading time (< 3 h/ ≥3 h per day) (40), and anabolic steroid use (yes/no). The level of physical activity was assessed according to the International Physical Activity Questionnaire–IPAQ (high, moderate, and low) (41).

In order to avoid possible bias due to loss to follow-up (74.0%) that occurred during the cohort period, weighting was performed by the inverse of the selection probability (participation in the follow-up). For that, the following baseline variables were used: type of birth (cesarean section/vaginal); sex (male/female); birth weight (< 2500 g / ≥2,500 g); parity (1 birth/ 2–4 births/ ≥5 births); prematurity (preterm birth < 37 weeks/ term birth ≥37 weeks); maternal age (< 20 years/20–34 years/> 34 years); maternal schooling (≤ 4 years of schooling/ 5–8 years of schooling/ 9–11 years of schooling/ ≥12 years of schooling); maternal smoking during pregnancy (yes/no); maternal marital status (with a partner/without a partner), number of prenatal consultations (0 consultations/1–5 consultations/ ≥6 consultations); maternal occupation (non-manual/skilled manual/unskilled manual); length at birth (< 50 cm/ ≥50 cm), and family income in quartiles.

### Data analysis

This study had as an exposure variable the proportion of consumption in grams of UPF at 23–25 years of age, obtained by the percentage of total daily intake (daily intake of UPF (g)/total daily intake of foods and beverages(g)^*^100). Outcomes were body adiposity (measured by BMI, BFP and LMI), distribution of body fat (GA, GG and AGR) and lean mass (MMP, LMI and ALMI), assessed at 37–39 years. All variables were treated continuously.

In order to establish the minimum set of adjustment variables, reduce confounder bias, collision bias and avoid the inclusion of unnecessary variables in multivariate analysis, a theoretical model was developed based on the construction of a Directed Acyclic Graph (DAG), through the online DAGitty (version 3.0) software (42), according to Supplementary Figure S2. The variables to compose the minimum adjustment set for confounders identified in the DAG by the backdoor criterion (42) were: age, skin color, family income, sex, marital status, level of physical activity, smoking, screen time, anabolic steroid use, and alcohol consumption. As in the FFQ, alcohol consumption was already taken into account within the groups, and it was decided not to use it as an adjustment variable. In addition, as analyzes were performed by % of UPF grams, the TDEI variable was included in the adjustment. All adjustment variables were considered at 23–25 years of age.

The variables used to analyze loss to follow-up were evaluated using the Chi-square test and, by means of logistic regression, a weight was generated for each participant, obtained by the inverse of the selection probability.

Statistical analyzes were performed using Stata^®^ (version 14.0) software (Stata Corporation, College Station, Texas, USA). Qualitative variables were described in absolute and relative frequencies and quantitative variables in means and standard deviations. To assess the normality of distribution of variables, asymmetry and kurtosis coefficients were used.

Sociodemographic and lifestyle variables were compared using Pearson's chi-square or Fisher's exact test. Means of total calorie consumption, food groups, macronutrients, fiber, alcohol, and body composition were compared between sexes using Student's *t*-test. The significance level was set at 0.05 and a 95% confidence interval was adopted.

Linear regression models were fitted to verify the association between UPF consumption and body composition measurements. Unadjusted and adjusted analyzes were performed for confounder variables identified in the DAG from the backdoor criterion. Analyzes were performed with linear UPF consumption variables and, later, quadratic terms were added to investigate non-linear effects. The presence of possible interactions (UPF consumption and sex) was also tested. For interactions, a 0.10 significance level was considered. Based on the adjusted regression models, in the presence of interaction, conditional effects were plotted for each body composition outcome according to UPF consumption, for each sex (43). These conditional effects are statistics calculated from predictions of a previously fitted model on fixed values of some covariates and mean or otherwise integrating over the remaining covariates (44).

All analyzes were also performed to verify the association between the UPF consumption caloric percentage (in % kcal) and body composition, whose results are shown in Supplementary Tables S2–S4 and Supplementary Figures S3–S8. For these analyses, adjustment was performed for the variables selected in the DAG, with the exception of alcohol consumption. Furthermore, the TDEI variable was included in the adjustment.

## Results

At 23–25 years of age, 50.8% were male, white (61.8%), without a partner (68.6%), non-smokers (82.9%), and with reading and TV time ≥3 h (66.5%). The most prevalent family income was < 5 minimum wages (34.4%). Almost half (49.2%) of the participants had a low level of physical activity, followed by moderate (29.9%) and high (20.5%) levels. Only 2.2% used anabolic steroids (Table 1).

**Table 1:** Sociodemographic characteristics and lifestyle habits of adults aged 23–25 years participating in the 1978/1979 Ribeirão Preto birth cohort study (São Paulo, Brazil, 2002–2004).

**Variable**	**Total**		**Male**		**Female**		*p*-value^**^
	***n***	%^*^	***n***	%^*^	***n***	%^*^	
Sex	1,021	100.0	481	50.8	540	49.3	-
**Age**							
23	315	30.5	146	29.2	169	31.8	0.688
24	503	49.0	237	49.9	266	48.0	
25	203	20.5	98	20.9	105	20.2	
**Race/Ethnic group**							
White	665	61.7	311	61.8	354	61.6	0.507
Brown	293	31.3	136	30.1	157	32.5	
Black	52	5.9	27	6.7	25	5.1	
Yellow/Oriental	11	1.1	7	1.4	4	0.8	
**Family income (in MW^a^)**							
< 5	324	34.4	138	31.4	186	37.5	< 0.001
5-9.9	329	30.9	147	29.7	182	32.0	
>9.9	395	26.7	168	32.3	127	21.0	
No information	73	8.0	28	6.6	45	9.5	
**Marital status**							
With a partner	314	31.4	121	26.1	193	36.9	< 0.001
Without a partner	707	68.6	360	73.9	347	63.1	
**Smoking**							
No	857	82.9	392	80.6	465	85.2	0.074
Yes	164	17.1	89	19.4	75	14.8	
**Physical activity level**							
Low	498	49.2	276	57.9	222	40.3	< 0.001
Moderate	311	29.9	137	27.5	174	32.3	
High	209	20.5	68	14.7	141	26.6	
No information	3	0.4	0	0.0	3	0.8	
**Television and reading time**							
< 3 h	343	33.5	165	34.1	178	32.9	0.714
≥3 h	678	66.5	316	65.9	362	67.1	
**Anabolic steroid use**							
No	999	97.8	461	96.0	538	99.7	< 0.001
Yes	22	2.2	20	4.0	2	0.3	

* Relative frequencies weighted by loss to follow-up.

** Pearson's chi-square or Fisher's exact test.

a MW: minimum wage, which from 2002 to 2004 ranged from R$ 200,00 to 260,00.

The average daily food consumption was 2,165.5g (±744.1 g), with 35.8% (±15.3%) coming from UPF, 7.2% (±5.7%) from processed foods, and 57.0 % (±15.2%) from food preparations. The mean UPF consumption (% grams) was 36.3% (±14.7%) among women and 35.3% (±15.8%) among men (Table 2).

**Table 2:** Food group consumption according to the NOVA classification and macronutrients and alcohol consumption by 23–25-year old participants (2002/2004) of the 1978/1979 Ribeirão Preto birth cohort study (São Paulo, Brazil).

**Energy/Food group/**		**All (*****n*** = **1,021)**		**Male (*****n*** = **481)**		**Female (*****n*** = **540)**		*p*-value^*^
**Macronutrients**		**Mean**	**SD**	**Mean**	**SD**	**Mean**	**SD**	
Total dietary energy intake	Kcal	2,254.1	692.8	2,457.0	702.1	2,073.3	632.2	< 0.001
Total consumption (in grams)	Grams/day	2,165.5	744.1	2,329.4	732.8	2,019.5	724.0	< 0.001
Ultra-processed foods	Diet Kcal	877.1	426.2	912.6	441.4	845.5	409.9	0.011
	% TDEI	38.1	11.3	36.1	10.9	39.9	11.3	< 0.001
	Grams/day	813.3	583.9	855.5	561.9	775.8	600.9	0.029
	% Grams/day	35.8	15.3	35.3	14.7	36.3	15.8	0.281
Processed foods	Diet Kcal	241.7	140.6	283.3	143.9	204.6	126.7	< 0.001
	% TDEI	10.8	5.3	11.7	5.2	9.9	5.2	< 0.001
	Grams/day	154.9	145.7	202.2	156.5	112.7	120.8	< 0.001
	% Grams/day	7.2	5.7	8.9	6.1	5.6	4.8	< 0.001
Food preparations^a^	Diet Kcal	1,132.5	385.1	1,258.7	396.6	1,020.2	337.3	< 0.001
	% TDEI	51.1	11.3	52.1	11.4	50.1	11.2	0.006
	Grams/day	1,197.3	445.6	1,271.7	460.1	1,131.1	421.8	< 0.001
	% Grams/day	57.0	15.2	55.9	14.6	58.1	15.7	0.020
Macronutrients and alcohol	Carbohydrates (g) /1000Kcal	139.8	16.4	136.8	16.0	142.6	16.2	< 0.001
	Lipids (g) /1000Kcal	28.8	5.4	29.3	5.3	28.4	5.5	0.010
	Proteins (g) /1000Kcal	42.2	7.9	43.0	7.4	41.4	8.2	0.001
	Fibers (g) /1000Kcal	11.1	2.9	10.7	2.7	11.4	3.0	< 0.001
	Alcohol (g) /1000kcal	1.8	2.6	2.5	2.8	1.2	2.2	< 0.001
	Carbohydrates^**^	55.9	6.5	54.7	6.4	57.0	6.5	< 0.001
	Proteins (%)^**^	16.9	3.2	17.2	3.0	16.6	3.3	0.001
	Lipids (%)^**^	25.9	4.9	26.4	4.8	25.6	4.9	0.011

TDEI, total dietary energy intake.

SD, Standard deviation.

* Student's t-test.

** The kilocalories from alcohol were included in the TDEI calculation, and then the final sum is not 100%.

a Group formed by Group 1 “Raw/minimally processed foods” plus Group 2 “Culinary ingredients”.

There was a high consumption of sugar-sweetened beverages (SSB) (24.65% of the total food items/beverage consumed per day) by the sample studied. After these, the most consumed UPF was snacks, dairy products and candies. The most consumed food items/beverages in each group and the percentage contribution to the total dietary intake (in % of grams and in % of caloric contribution) are listed in Supplementary Tables S1, S2, respectively.

At 37–39 years of age, women had higher total body adiposity (BFP, FMI), distributed in the gynoid region, while men had higher BMI, and greater LM (LMP, LMI, ALMI) and AF (Table 3).

**Table 3:** Mean of body composition measurements at 37–39 years (2016/2017) according to sex of participants of the 1978/1979 Ribeirão Preto birth cohort study (São Paulo, Brazil).

**Body composition measurements**	**General mean** ±**SD**		*p*-value^*^
	**Male**	**Female**	
	**(*n* = 481)**	**(*n* = 540)**	
Body mass index (kg/m^2^)	28.9 ± 4.4	28.1 ± 5.6	0.006
Body fat percentage (%)	26.4 ± 7.7	37.9 ± 8.3	< 0.001
Fat mass index (kg/m^2^)	7.9 ± 3.4	11.1 ± 4.3	< 0.001
Android fat (kg)	2.6 ± 1.0	2.3 ± 1.1	< 0.001
Gynoid fat (kg)	4.7 ± 1.6	6.0 ± 1.7	< 0.001
Android/gynoid fat ratio	0.4 ± 0.2	0.02 ± 0.1	0.323
Lean fat percentage (%)	66.8 ± 6.5	54.9 ± 7.5	< 0.001
Lean mass index (kg/m^2^)	18.7 ± 1.7	14.7 ± 1.8	< 0.001
Appendicular lean mass index (kg/m^2^)	8.8 ± 0.9	6.4 ± 0.8	< 0.001

* Student's t-test. SD, Standard deviation.

In the analysis in % grams, a longitudinal association was observed between UPF consumption and FMI (crude analysis: β = 0.02; 95%CI 0.00, 0.04; *p* = 0.017, and adjusted β = 0.02; 95%CI 0.00, 0.04; *p* = 0.022); and between UFP consumption and BFP (crude analysis: β = 0.05; 95%CI 0.00, 0.09; *p* = 0.019, and adjusted β = 0.04; 95%CI 0.00, 0.08; *p* = 0.012) (Table 4).

**Table 4:** Crude and adjusted linear regression analysis between ultra-processed foods consumption (% grams) at 23–25 years (2002/2004) and body composition measurements at 37–39 years (2016/2017) in participants of the 1978/1979 Ribeirão Preto birth cohort study (São Paulo, Brazil).

**Body composition measurements**	**% Of ultra-processed foods grams**			
	**Crude analysis**		**Adjusted analysis^d^**	
	**β (95%CI)**	***p*-value**	**β (95%CI)**	***p*-value**
**Body mass index (kg/m** ^**2**^ **)**				
a	0.02 (0.00, 0.04)	0.143	0.02 (0.00, 0.04)	0.135
b			−1.15 (−1.84, −0.45)	0.001
c	0.04 (0.00, 0.08)	0.064	0.04 (0.00, 0.08)	0.058
**Fat mass index (kg/m2)**				
a	0.02 (0.00, 0.04)	0.017	0.02 (0.00, 0.04)	0.022
b			2.79 (2.27, 3.32)	< 0.001
c	0.04 (0.00, 0.07)	0.029	0.04 (0.00, 0.07)	0.021
**Body fat percentage (%)**				
a	0.05 (0.00, 0.09)	0.019	0.04 (0.00, 0.08)	0.012
b			10.54 (9.47, 11.63)	< 0.001
c	0.09 (0.02, 0.15)	0.016	0.09 (0.02, 0.16)	0.010
**Android fat (kg)**				
a	0.002 (−0.002, 0.007)	0.338	0.003 (−0.001, 0.008)	0.512
b			−0.32 (−0.48, −0.17)	0.004
c	0.01 (0.00, 0.02)	0.007	0.01 (0.00, 0.02)	0.005
**Gynoid fat (kg)**				
a	0.006 (−0.003, 0.01)	0.165	0.006 (−0.002, 0.01)	0.147
b			1.18 (0.93, 1.43)	< 0.001
c	0.02(0.00, 0.04)	0.015	0.02 (0.00, 0.04)	0.017
**Android/gynoid fat ratio**				
a	0.0002 (−0.0004, 0.0008)	0.597	0.0001 (−0.0004, 0.0006)	0.617
b			−0.17 (−0.19, −0.15)	< 0.001
c	0.0005 (−0.0005, 0.001)	0.348	0.0006 (−0.0004, 0.002)	0.225
**Lean mass percentage (%)**				
a	−0.03 (−0.07, 0.02)	0.197	−0.02 (−0.06, 0.00)	0.173
b			−10.59 (−11.60, −9.58)	< 0.001
c	−0.10 (−0.16, −0.03)	0.003	−0.10 (−0.16, −0.03)	0.002
**Lean mass index (kg/m** ^**2**^ **)**				
a	−0.005 (−0.02, 0.007)	0.447	−0.004 (−0.01, 0.00)	0.373
b			−3.75 (−4.00, −3.49)	< 0.001
c	0.004 (−0.01, 0.02)	0.650	0.00 (−0.01, 0.02)	0.695
**Appendicular lean mass index (kg/m** ^**2**^ **)**				
a	−0.004 (−0.01, 0.00)	0.273	−0.003 (−0.007, 0.001)	0.168
b			−2.20 (−2.32, −2.07)	< 0.001
c	0.00 (−0.007, 0.008)	0.916	0.00 (−0.008, 0.008)	0.925

a β coefficient of linear regression.

b β coefficient of linear regression for women.

c β coefficient of the interaction term between consumption of ultra-processed foods and sex.

d Analysis adjusted for sex, age, family income, marital status, television and reading time, physical activity level, smoking, anabolic steroid use, and total dietary energy intake.

There was interaction between sex and UPF consumption for the BMI (*p* = 0.058), FMI (*p* = 0.021), BFP (*p* = 0.010), AF (*p* = 0.005), GF (*p* = 0.017) and LMP (*p* = 0.002) variables (Table 4). Therefore, the results of the linear regressions of the association between UPF consumption and these variables were calculated conditional effects for each sex (Table 5). In the crude analysis, there was an association between UPF consumption and increased BMI (β = 0.04; 95%CI 0.01, 0.07; *p* = 0.024), FMI (β = 0.04; 95%CI 0.01,0.06; *p* = 0.003), BFP (β = 0.08; IC95% 0.03, 0.13; *p* = 0.001), AF (β = 0.01; IC95% 0.00, 0.01; *p* = 0.007), GF (β = 0.01; IC95% 0.00, 0.02; *p* = 0.021), and decrease in LMP (β = −0.06; 95%CI −0.11, −0.02; *p* = 0.004), only in women. In the adjusted analysis, a 10% increase in the percentage of UPF consumption (in grams) was associated with a longitudinal increase of 0.4 kg/m^2^ in BMI (β = 0.04; 95%CI 0.01, 0.07; *p* = 0.023), 0, 4 kg/m^2^ of LMI (β = 0.04; 95%CI 0.01,0.06; *p* = 0.002), 0.8% in BFP (β = 0.08; IC95% 0.04, 0.13; *p* = < 0.001), 0.1 kg of AF (β = 0.01; 95%CI 0.00,0.01; *p* = 0.003), 0.1 kg of GF (β = 0.01; 95%CI 0.00, 0.02; *p* = 0.011), and a 0.7% decrease in LMP (β = −0.07; 95%CI −0.11, −0.03; *p* = 0.001), only in women (Table 5 and Supplementary Figures S3–S8).

**Table 5:** Conditional effects of ultra-processed foods consumption (% grams) according to sex at 23–25 years (2002/2004) and body composition measurements at 37–39 years (2016/2017) in participants of the 1978/1979 Ribeirão Preto birth cohort study (São Paulo, Brazil).

**Linear regression prediction of body composition measurements**	**% Of ultra-processed foods grams**			
	**Crude analysis**		**Adjusted analysis^a^**	
	**β (95%CI)**	***p*-value**	**β (95%CI)**	***p*-value**
**Body mass index (kg/m** ^**2**^ **)**				
Men	0.00 (−0.03, 0.03)	0.758	0.00 (−0.04,0.03)	0.725
Women	0.04 (0.01, 0.07)	0.024	0.04 (0.01, 0.07)	0.023
**Fat mass index (kg/m** ^**2**^ **)**				
Men	0.00 (−0.02, 0.02)	0.967	0.00 (−0.03;0.02)	0.920
Women	0.04 (0.01, 0.06)	0.003	0.04 (0.01, 0.06)	0.002
**Body fat percentage**				
Men	0.00 (−0.06, 0.05)	0.862	0.00 (−0.05, 0.05)	0.870
Women	0.08 (0.03, 0.13)	0.001	0.08 (0.04, 0.13)	< 0.001
**Android fat (kg)**				
Men	0.00 (−0.01, 0.00)	0.193	0.00 (−0.01, 0.00)	0.226
Women	0.01 (0.00, 0.01)	0.007	0.01 (0.00, 0.01)	0.003
**Gynoid fat (kg)**				
Men	−0.01 (−0.02, 0.00)	0.269	−0.01 (−0.02, 0.00)	0.366
Women	0.01 (0.00, 0.02)	0.021	0.01 (0.00, 0.02)	0.011
**Lean mass percentage (%)**				
Men	0.04 (−0.01, 0.08)	0.144	0.03 (−0.02, 0.08)	0.173
Women	−0.06 (−0.11, −0.02)	0.004	−0.07 (−0.11, −0.03)	0.001

a Analysis adjusted for sex, age, family income, marital status, television and reading time, physical activity level, smoking, anabolic steroid use and total dietary energy intake.

In analyzes in % of caloric contribution, the longitudinal association was observed only in the crude analyzes for the FMI (*p* = 0.001), BFP (*p* < 0.001), GF (*p* = 0.031), AGR (*p* = 0.021), LMP (*p* = 0.003), LMI (*p* = 0.003) and ALMI (*p* = 0.001) variables. After adjustments, significance was maintained only for the BFP variable (*p* = 0.042) (Supplementary Table S3). There was interaction between sex and UPF consumption for the BMI (*p* = 0.038), FMI (*p* = 0.018), BFP (*p* = 0.017), AF (*p* = 0.071), GF (*p* = 0.038) and LMP (*p* = 0.004) variables (Supplementary Table S3). In the analysis of these conditional effects by sex, the crude analysis showed an association between UPF consumption and increased BMI (β = 0.05; 95%CI 0.00, 0.09; *p* = 0.032), FMI (β = 0.04; 95%CI 0.01, 0.08).; *p* = 0.008), BFP (β = 0.09; 95%CI 0.02, 0.16; *p* = 0.007), and decrease in LMP (β = −0.07; 95%CI −0.13, −0.004; *p* = 0.038), only in women. In the adjusted analysis, the 10% increase in the UPF consumption percentage was associated with a longitudinal increase of 0.5 kg/m^2^ in BMI (β = 0.05; 95%CI 0.01, 0.09; *p* = 0.016), 0.5 kg/m^2^ in FMI (β = 0.05; 95%CI 0.02, 0.08; *p* = 0.003), 1.1% in BFP (β = 0.11; 95%CI 0.04, 0.17; *p* = 0.002), and 0.8% decrease in LMP (β = −0.08; 95%CI −0.14, −0.02; *p* = 0.006), only in women (Supplementary Tables S3, S4 and Supplementary Figures S3–S8).

Quadratic terms of UPF consumption (in %grams and in % caloric contribution) were tested for all body composition variables, but there was no statistical significance.

## Discussion

This study investigated the association between the UPF contribution percentage and the diet and body composition assessed by high validity methods in adults of a cohort study. The greatest UPF contribution to the diet was associated with an increase in fat and a decrease in lean mass years later, only among women. These findings demonstrate that a higher UPF consumption has a negative impact on the body composition of women in the long term, contributing to increasing the prevalence of overweight, which is also a risk factor for the emergence of other NCDs.

As limitations, we point out the use of the FFQ, a method that is subject to memory and measurement bias due to not counting the consumption of food items that are not listed, tending to overestimate food intake. In addition, the FFQ used was not originally constructed aimed at identifying food consumption according to the degree of processing, given that data collection took place in a period before the emergence of the NOVA classification, and some different food groups were placed in the same question. To minimize this limitation, adjustments were made, considering the estimates of food consumption for the state of São Paulo at the time of data collection (37). We reiterate, however, that the instrument used was adapted for use in NCD prevention programs aimed at adults, and was validated (32), reflecting the food consumption of the time, and although there are some limitations, the FFQ is a widely used method in epidemiological studies due to its low cost, practicality, ability to assess the usual diet without changing the pattern of food consumption (45), as long as the methodological aspects for its elaboration are carefully planned, ensuring data reliability and accuracy (45).

Another limitation was the use of the screen time variable, considering that the database only had information about TV time and reading in aggregate form. We emphasize that at the time of data collection (2002/2004), the use of screens such as computers, smartphones and video games, among others, was not yet so widespread in Brazil, therefore not appearing in the large population surveys carried out in the country until then (40). Finally, another limitation of this study involved the losses to follow-up. Inverse probability weighting was used to minimize the effects of these losses on analysis.

As strengths, we highlight the fact that longitudinal studies that evaluated the body composition of adults with high validity methods are still scarce and, to date, there are no studies that have demonstrated associations between UPF consumption and the body composition of adults, especially the influence of UPF consumption on body lean mass.

The UPF contribution percentage in grams in the diet of adults in Ribeirão Preto was 35.8% (equivalent to 38.1% of TDEI). This represents practically double the Brazilian average (19.7% of TDEI), according to the 2017–2018 Household Budget Survey (POF) (19), which can be explained by the fact that the municipality is at more advanced levels of nutritional transition compared to other regions of the country, characterized by high caloric intake, high consumption of foods with low nutritional quality, and a sedentary lifestyle. Furthermore, young individuals, in the age group of the population of this study, tend to consume high levels of UPF in Brazil (46). A high UPF consumption in the diet of adults has been evidenced by several studies, indicating a high risk of developing NCDs due to the unbalanced nutritional composition of these products (14–22).

A highlight is the high ultra-processed SSB (soft drinks and industrialized juices) consumption, corresponding, in grams, to practically 14 of the total of food items/beverages consumed/day (24.7%), which is equivalent to 10.24% in caloric contribution percentage (Supplementary Tables S1, S2). High SSB consumption is associated with potential hormonal dysregulation, insulin resistance, dyslipidemia, increased adiposity and obesity, and is also related to obesogenic behaviors, such as sedentary lifestyle and high screen time (47, 48), lifestyle habits that have been common in the population of this study. In addition, a higher SSB consumption (but not the general UPF group) was associated with higher urinary concentrations of bisphenol A (BPA), an endocrine disruptor that is associated with increased prevalence of risk factors for abdominal obesity, diabetes and hypertension (48, 49).

The pattern of body composition found in this research, where women had higher levels of total adiposity and GF, and men had higher means of BMI, AF, AGR and lean mass was also found in other ethnic groups (38). This pattern can be explained due to biological differences (50, 51), represents a higher risk of sarcopenic obesity for women (51) and a higher risk of cardiovascular diseases and metabolic syndrome (52) for men considering that body fat distribution has a greater impact on cardio-metabolic risk than excess total adiposity (51).

Despite this pattern of body distribution, our results showed a deleterious effect of UPF only on the body composition of women, corroborating other evidence that associates UPF with excess body weight and consequent higher adiposity (20–22, 53–57), especially in women. In another Brazilian study, the authors found a positive association between UPF consumption and overweight and obesity in women, but not in men (21). In Switzerland, UPF was associated with excess body weight only in women (54). In South Korea, UPF was also associated with obesity only among women (55). In the United States, Juul et al. (20) observed significant positive associations between UPF consumption and overweight or obesity in both sexes, but with a more pronounced association among women, a result also found in the United Kingdom (20, 22).

Scientific evidence suggests that UPF promotes changes in body composition through a range of mechanisms, such as the unbalanced nutritional composition of foods and beverages, which favors increased energy and nutrient consumption and ingredients of low nutritional quality, such as refined sugars (11–18); the presence of food additives and contaminants produced during processing, which alter the profile and composition of the intestinal microbiota (56), and changes in the food matrix induced by food processing that seem to influence the kinetics of nutrient absorption and alterations in gut-brain satiety signaling (48, 53), among other mechanisms not yet clarified that interrelate, promoting inflammation, oxidative stress and consequent weight gain (48). The effects of these mechanisms appear to affect the sexes differently, with more impact on women. UPF is associated with a higher glycemic response, and women appear to be more sensitive to the UPF hyperglycemic effect than men (48). Besides, changes in the intestinal microbiota caused by UPF seem to affect men and women differently (56).

As for the distribution of body fat, a higher UPF consumption favored the increase in AF and GF, but not in AGR, also only among women. Despite the non-association with AGR, which is an indicator of abdominal obesity, UPF is found to be more harmful to women with regard to the association with an increase in AF, which is associated with a greater risk of cardiovascular outcomes and metabolic syndrome (28, 52). We emphasize that this association for AF may not have been found in the analysis in the UPF caloric contribution percentage due to the fact that the analysis in grams takes into account foods and beverages widely consumed by the population, which have a high content of additives and low caloric contribution, such as SSB, especially diet and light soft drinks.

An association between UPF consumption and a decrease in LMP was demonstrated only in women. Although no association was found for LMI and ALMI, which are indices relativized by height are commonly associated with the diagnosis of sarcopenia, especially the latter, these results are worrying given the importance of LM in maintaining the population's health and quality of life. LM provides useful information about an individual's health and nutrition, playing an important role in maintaining bone density, preserving strength, reducing the risk of injuries and falls, and improving metabolism and general health. Reduction in LM with age or due to sarcopenia is associated with decreased function and quality of life and the frailty development (57, 58). Thus, although the reduction in LM was not detected by the indices generally used in the diagnosis of sarcopenia, the decrease in LMP already points to a deleterious effect of UPF on LM, not having been detected by LMI and ALMI because this population is still found at the peak of the LM. Furthermore, this pattern of body distribution characterized by high adiposity and decreased LM in women points to the development of sarcopenic obesity, which makes them more prone to the development of diabetes mellitus, abdominal obesity, and an increased risk of death (59).

## Conclusion

Our results indicate that the high UPF contribution percentage to the diet of Brazilian adults is associated with a longitudinal increase in FM and a decrease in LM only among women, favoring a deleterious composition pattern for females.

## Data availability statement

The original contributions presented in the study are included in the article/Supplementary material, further inquiries can be directed to the corresponding author/s.

## Ethics statement

The studies involving human participants were reviewed and approved by Research Ethics Committee (CEP) of the University Hospital of FMRP-USP School of Medicine. The patients/participants provided their written informed consent to participate in this study.

## Author contributions

LR performed material preparation and data analysis and wrote the first draft of the manuscript. All authors commented on the other versions, contributed to the study conception and design, read, and approved the final manuscript.

## Funding

This research was funded by the Coordination for the Improvement of Higher Education Personnel-Brazil (CAPES), the National Council for Scientific and Technological Development -CNPq (grant number 400943/2013-1), the Research Support Foundation of the State of São Paulo -FAPESP (grant number 00/09508-7) and the Teaching, Research, and Assistance Support Foundation (FAEPA).

## Conflict of interest

The authors declare that the research was conducted in the absence of any commercial or financial relationships that could be construed as a potential conflict of interest.

## Publisher's note

All claims expressed in this article are solely those of the authors and do not necessarily represent those of their affiliated organizations, or those of the publisher, the editors and the reviewers. Any product that may be evaluated in this article, or claim that may be made by its manufacturer, is not guaranteed or endorsed by the publisher.

## Abbreviations

ADP: Air displacement plethysmography
AG: Android fat
AGR: Android-gynoid fat ratio
ALMI: Appendicular lean mass index
BPA: Bisphenol A
BFP: Body fat percentage
BMI: Body mass index
NCDs: Chronic noncommunicable diseases
DXA: Dual-energy R-ray absorptiometry
FM: Fat mass
FMI: Fat mass index
FFM: Fat-free mass
FFQ: Food frequency questionnaire
GF: Gynoid fat
LM: Lean mass
LMI: Lean mass index
LMP: Lean mass percentage
MM: Muscle mass
POF: Household Budget Survey
SSB: Sugar-sweetened beverages
TDEI: Total dietary energy intake
UPF: Ultra-processed food.
