# Supplementary material for: Ultra-processed food consumption is associated with increase in fat mass and decrease in lean mass in Brazilian women: A cohort study
Source: Front Nutr. 2022 Oct 14;9:1006018. doi: 10.3389/fnut.2022.1006018 (PMC9615037; doi:10.3389/fnut.2022.1006018)
Supplement: Supplementary file 1 [file Data_Sheet_1.pdf]

Supplementary Material

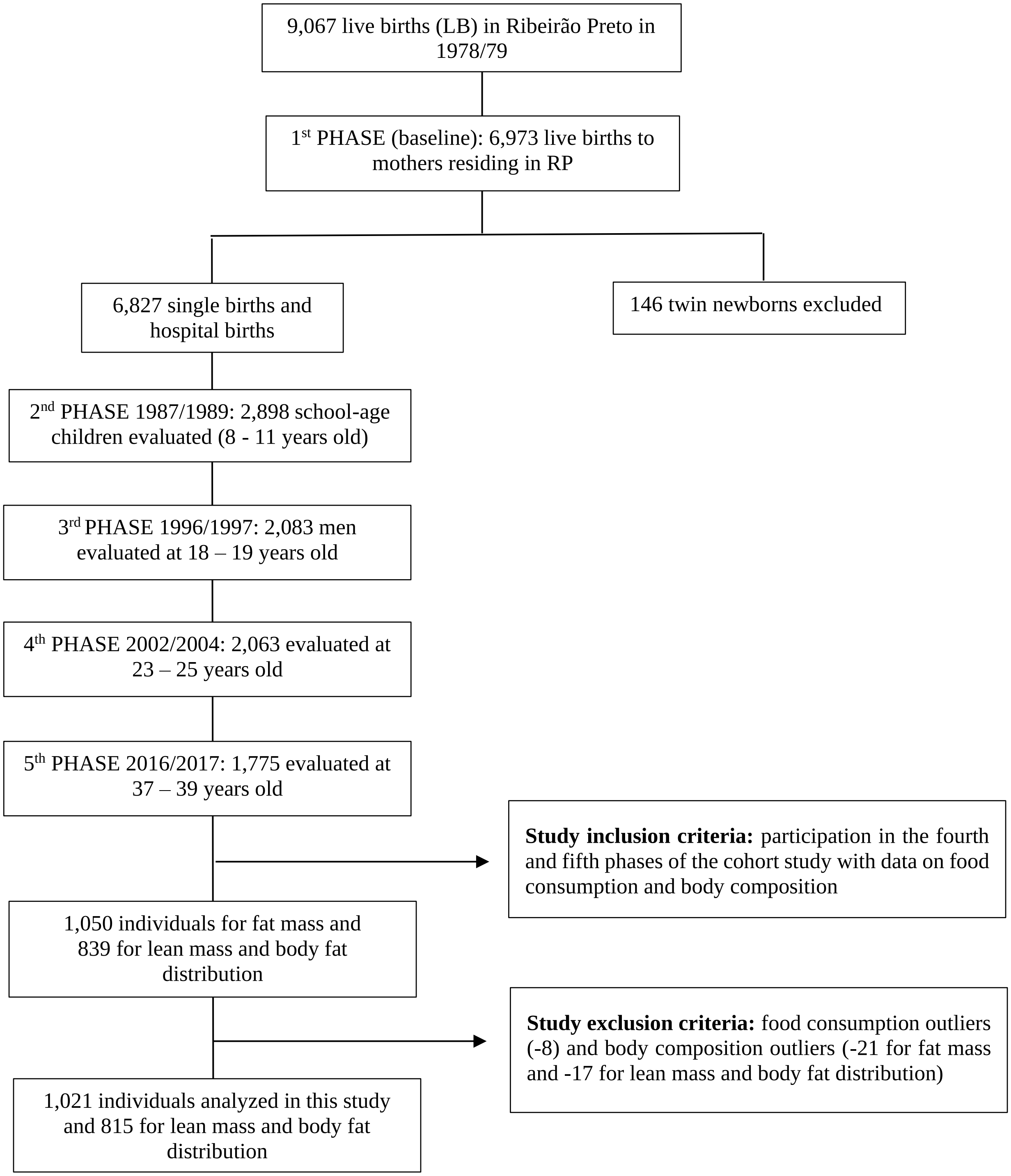

Figure S1. Flowchart of birth cohort study participants from Ribeirão Preto, São Paulo, Brazil (1978/79-2016/17)

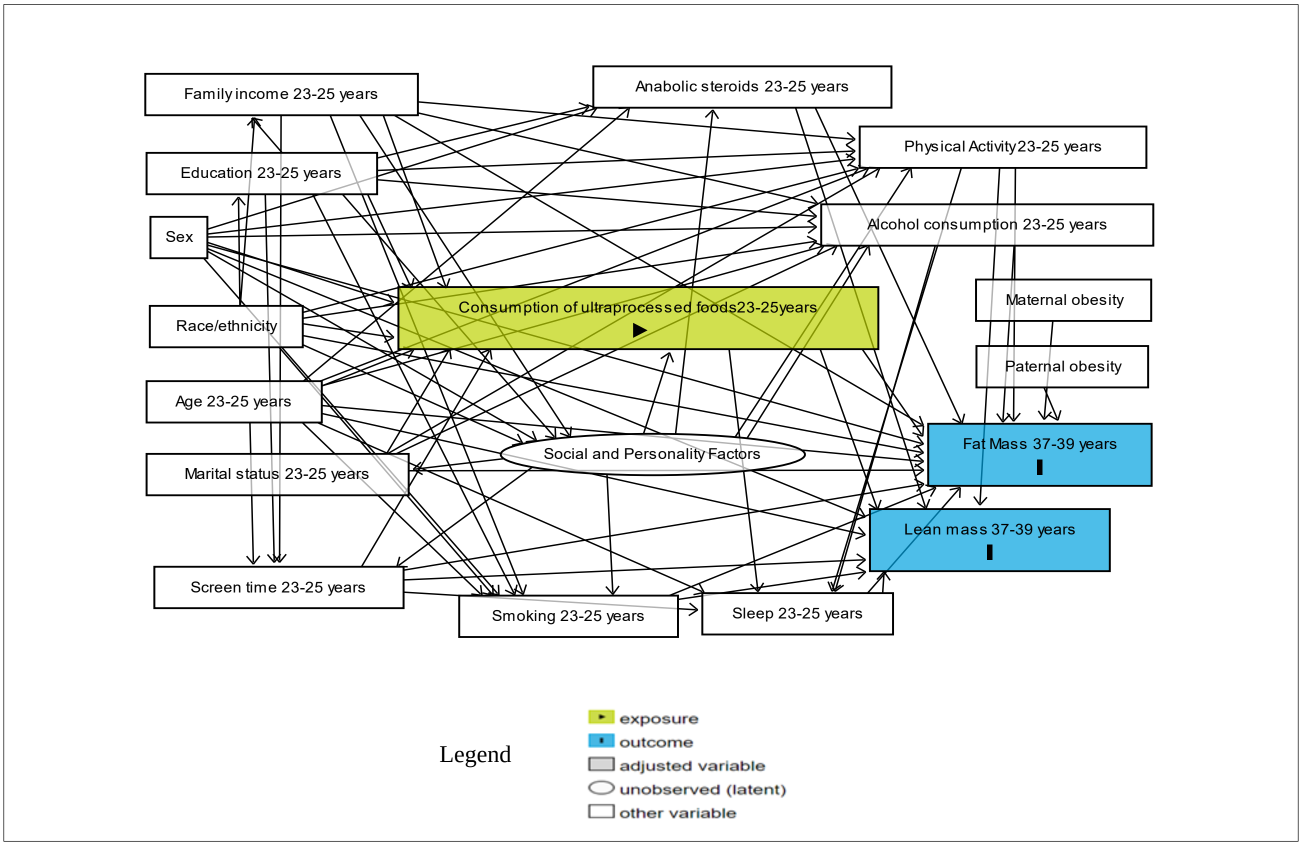

Figure S2. Directed acyclic graphs of the effect of ultra-processed foods consumption at 23-25 years on body adiposity, and lean mass of adults at 37-39 years

Table S1. Gram contribution percentage of food preparations, processed and ultra-processed foods to the total dietary energy intake by adults aged 23-25 years (2002/2004) participating in the 1978/1979 Ribeirão Preto birth cohort study (São Paulo, Brazil)

Good groups<br>Food items	Consumption mean in % grams						p-value[a]
	All (n=1,021)	Standard deviation	Men (n=481)	Standard deviation	Women (n=540)	Standard deviation	
**ULTRA-PROCESSED FOODS**							
Sugar-sweetened beverages[b]	24.64	15.20	24.45	14.06	24.82	16.16	0.697
Savory snacks	3.57	3.29	4.07	3.47	3.04	2.93	< 0.001
Dairy products	1.45	1.98	1.09	1.48	1.76	2.30	< 0.001
Candies	1.34	1.27	1.16	1.18	1.49	1.34	< 0.001
Cold meats	1.11	0.94	1.20	0.95	1.03	0.93	0.005
Cake	0.87	1.36	0.70	0.90	1.02	1.65	< 0.001
Ultra-processed bread	0.80	0.71	0.72	0.64	0.85	0.77	0.005
Crackers	0.74	0.89	0.58	0.68	0.87	1.03	< 0.001
Instant noodles	0.43	0.35	0.43	0.35	0.44	0.35	0.567
Snacks	0.38	0.73	0.32	0.79	0.43	0.67	0.018
Mayonnaise	0.18	0.28	0.20	0.27	0.16	0.29	0.029
Granola	0.16	0.39	0.13	0.34	0.18	0.43	0.050
Margarine	0.15	0.19	0.13	0.14	0.17	0.22	< 0.001
Distilled drinks	0.06	0.17	0.08	0.21	0.04	0.12	< 0.001
**PROCESSED FOODS**							
Fermented drinks	3.83	5.37	5.45	5.89	2.40	4.39	< 0.001
Bread	2.40	1.70	2.49	1.65	2.31	1.75	0.099
Cheese	0.68	0.69	0.67	0.69	0.70	0.68	0.435
Salty-cured meats	0.12	0.23	0.16	0.26	0.09	0.18	< 0.001
Fruit dessert	0.09	0.26	0.08	0.29	0.09	0.23	0.489
Jam	0.01	0.04	0.01	0.04	0.01	0.04	0.770
**FOOD PREPARATIONS**							
Fruit and fruit juices	14.19	9.34	12.79	8.73	15.44	9.68	< 0.001
Rice	8.43	4.72	8.90	4.76	8.03	4.65	0.004
Milk and yogurt	7.11	7.13	7.10	6.85	7.12	7.38	0.986
Legumes	6.36	4.52	7.14	4.27	5.67	4.63	< 0.001
Vegetables	5.78	4.23	4.63	3.57	6.79	4.50	< 0.001
Coffee/tea	4.93	6.12	4.84	6.38	5.01	5.88	0.661
Read meat	3.86	2.44	4.08	2.32	3.66	2.53	0.006
Potato, cassava and corn	1.90	1.43	1.77	1.31	2.00	1.53	0.011
Chicken/poultry	1.67	1.43	1.62	1.45	1.72	1.42	0.238
Pasta	1.17	0.91	1.22	0.94	1.13	0.88	0.111
Eggs	0.61	0.70	0.72	0.77	0.52	0.61	< 0.001
Fish	0.56	0.81	0.55	0.82	0.57	0.80	0.631
Popcorn	0.19	0.36	0.16	0.39	0.21	0.33	0.018
Chestnuts and oilseeds	0.12	0.40	0.18	0.49	0.08	0.29	< 0.001
Offal	0.10	0.27	0.12	0.30	0.09	0.24	0.114
Butter	0.04	0.10	0.04	0.09	0.04	0.12	0.625
Oatmeal	0.02	0.05	0.02	0.04	0.03	0.05	0.050
Honey	0.02	0.08	0.02	0.07	0.02	0.08	0.770

[a]Student's t-test

[b]Soft drinks and industrialized juices

Table S2. Percentage contribution of food preparations, processed, processed and ultra-processed foods to the total dietary energy intake by adults aged 23-25 years (2002/-2004) participating in the 1978/1979 Ribeirão Preto birth cohort study (São Paulo, Brazil)

Food groups<br>Food items	Mean percentage of % caloric contribution						p-value[a]
	All (n=1,021)	Standard deviation	Men (n=481)	Standard deviation	Women (n=540)	Standard deviation	
**ULTRA-PROCESSED FOODS**							
Sugar-sweetened beverages[b]	10.24	7.98	9.69	6.91	10.73	8.81	0.038
Snacks	8.16	6.89	9.33	7.39	7.11	6.24	< 0.001
Candies	4.29	3.96	3.57	3.50	4.94	4.23	< 0.001
Crackers	3.09	3.43	2.47	2.67	3.64	3.91	< 0.001
Ultra-processed bread	2.08	1.83	1.85	1.51	2.27	2.06	< 0.001
Cake	1.96	2.80	1.57	1.92	2.30	3.36	< 0.001
Cold meats	1.86	1.71	2.01	1.71	1.72	1.71	0.007
Snacks	1.71	2.64	1.42	2.48	1.97	2.74	0.001
Dairy products	1.63	2.15	1.23	1.62	1.98	2.49	< 0.001
Instant noodles	1.03	0.77	0.99	0.77	1.05	0.77	0.281
Margarine	0.85	1.05	0.75	0.88	0.94	1.18	0.005
Granola	0.58	1.44	0.46	1.20	0.68	1.63	0.017
Mayonnaise	0.52	0.77	0.58	0.79	0.46	0.75	0.011
Distilled drinks	0.14	0.43	0.19	0.52	0.09	0.32	0.004
**PROCESSED FOODS**							
Bread	6.13	4.05	6.35	3.90	5.95	4.18	0.117
Cheese	2.00	1.93	1.90	1.95	2.10	1.92	0.316
Fermented drinks	1.70	2.52	2.34	2.64	1.12	2.26	< 0.001
Salty-cured meats	0.63	1.12	0.81	1.27	0.46	0.94	< 0.001
Fruit dessert	0.23	0.70	0.22	0.78	0.25	0.62	0.514
Jam	0.04	0.12	0.03	0.12	0.04	0.12	0.584
**FOOD PREPARATIONS**							
Rice	9.90	5.41	10.4	5.55	9.46	5.25	0.006
Read meat	8.43	5.26	9.05	5.22	7.87	5.25	< 0.001
Fruit and fruit juices	8.15	5.56	7.31	5.19	8.90	5.76	< 0.001
Legumes	4.80	3.28	5.40	3.16	4.27	3.30	< 0.001
Milk and yogurt	3.94	4.04	4.01	4.00	3.87	4.08	0.586
Chicken/poultry	3.13	2.69	2.99	2.67	3.25	2.69	0.130
Pasta	2.96	2.16	3.12	2.32	2.82	1.99	0.028
Potato, cassava and corn	2.56	2.02	2.46	1.78	2.65	2.21	0.133
Coffee/tea	1.67	2.75	1.88	3.03	1.49	2.50	0.024
Vegetables	1.43	1.25	1.12	1.03	1.72	1.37	< 0.001
Eggs	1.08	1.20	1.26	1.29	0.91	1.09	< 0.001
Popcorn	0.84	1.30	0.70	1.22	0.97	1.35	0.001
Fish	0.80	1.19	0.76	1.16	0.83	1.22	0.362
Chestnuts and oilseeds	0.65	1.90	0.92	2.35	0.4	1.33	< 0.001
Butter	0.36	1.01	0.36	0.87	0.37	1.11	0.806
Offal	0.21	0.59	0.24	0.65	0.19	0.53	0.122
Oatmeal	0.08	0.20	0.06	0.16	0.09	0.22	0.017
Honey	0.07	0.23	0.06	0.23	0.07	0.23	0.585

[a]Student's t-test
[b]Soft drinks and industrialized juices

Table S3. Crude and adjusted linear regression analysis between ultra-processed food consumption (% TDEI) at 23-25 years (2002/2004) and body composition measurements at 37-39 years (2016/2017) in participants of the 1978/1979 Ribeirão Preto birth cohort study (São Paulo, Brazil)

**BODY COMPOSITION MEASUREMENTS**	**% TDEI OF ULTRA-PROCESSED FOODS**			
	**Crude analysis**		**Adjusted analysis [d]**	
	**β (95%CI)**	**p-value**	**β (95%CI)**	**p-value**
**Body mass index (kg/m²)**				
a	0.01 (-0.02, 0.04)	0.400	0.02 (-0,009, 0.05)	0.176
b			-1.23 (-1.94, -0.51)	0.001
c	0.06 (-0.00, 0.12)	0.056	0.06 (0.00, 0.12)	0.038
**Fat mass index (kg/m²)**				
a	0.04 (0.02, 0.06)	0.001	0.02 (-0,004, 0.05)	0.054
b			2.71 (2.17, 3.25)	< 0.001
c	0.05 (0.00, 0.09)	0.033	0.06 (0.01, 0.10)	0.018
**Body fat percentage (%)**				
a	0.11 (0.06, 0.17)	< 0.001	0.05 (0.00, 0.10)	0.042
b			10.36 (9.23, 11.49)	< 0.001
c	0.10 (0.00, 0.20)	0.032	0.11 (0.02, 0.21)	0.017
**Android fat (kg)**				
a	0.00 (-0,007, 0,005)	0.757	0,002 (-0.005, 0.008)	0.645
b			-0.33 (-0.49, -0.17)	< 0.001
c	0.01 (0.00, 0.04)	0.088	0.01 (0.00, 0.02)	0.071
**Gynoid fat (kg)**				
a	0.01 (0.00, 0.02)	0.031	0.00 (-0.01, 0.01)	0.764
b			1.34 (1.08, 1.60)	< 0.001
c	0.02 (0.00, 0.04)	0.029	0.02 (0.00, 0.04)	0.038
**Android/gynoid fat ratio**				
a	-0.0009 (-0,002, -0.0001)	0.021	0.00 (-0.0008, 0.0006)	0.843
b			-0.17 (-0.19, -0.15)	< 0.001
c	0.00 (-0.001, 0.001)	0.862	0.0004 (-0.001,0.002)	0.556
**Lean mass percentage (%)**				
a	-0.09 (-0.14, -0.03)	0.003	-0.02 (-0.07, 0.02)	0.390
b			-11.27 (-12.39, -10.16)	< 0.001
c	-0.11 (-0.19, -0.02)	0.018	-0.13 (-0.23, -0.04)	0,004
**Lean mass index (kg/m²)**				
a	-0.02 (-0.04, -0,008)	0.003	-0.003 (-0.02, 0.01)	0.586
b			-3.74 (-4.01, -3.46)	< 0.001
c	0.00 (-0.01, 0.03)	0.480	0.01 (-0.01, 0.03)	0.392
**Appendicular lean mass index (kg/m²)**				
a	-0.01 (-0.02, -0.006)	0.001	-0.003 (-0.009, 0.003)	0.398
b			-2.19 (-2.32, -2.06)	< 0.001
c	0.00 (-0.009, 0.01)	0.740	0.002 (-0.009, 0.01)	0.713

[a] β coefficient of linear regression
[b] β coefficient of linear regression for women
[c] β coefficient of the interaction term between consumption of ultra-processed foods and sex
[d] Analysis adjusted for sex, age, family income, marital status, television and reading time, physical activity level, smoking, anabolic steroid use and total dietary energy intake
TDEI: Total Dietary Energy Intake

Table S4. Conditional effects between the consumption of ultra-processed food consumption (% TDEI) according to sex at 23-25 years (2002/2004) and body composition measurements at 37-39 years (2016/2017) in participants in the 1978/1979 Ribeirão Preto birth cohort study (São Paulo, Brazil)

**Linear Regression Prediction of Body Composition Measurements**	**% TDEI OF ULTRA-PROCESSED FOODS**			
	**Crude analysis**		**Adjusted analysis [a]**	
	**β (95%CI)**	**p-value**	**β (95%CI)**	**p-value**
**Body mass index (kg/m²)**				
Men	-0.01 (-0.06,0.03)	0.593	-0.01 (-0.06,0.03)	0.602
Women	0.05 (0.00,0.09)	0.032	0.05 (0.01,0.09)	0.016
**Fat mass index (kg/m²)**				
Men	-0.01 (-0.04,0.03)	0.741	-0.01 (-0.04,0.03)	0.738
Women	0.04 (0.01,0.08)	0.008	0.05 (0.02,0.08)	0.003
**Body fat percentage**				
Men	-0.01 (-0.08,0.06)	0.704	-0.01 (-0.08,0.06)	0.790
Women	0.09 (0.02,0.16)	0.007	0.11 (0.04,0.17)	0.002
**Android fat (kg)**				
Men	0.00 (-0.02,0.00)	0.267	-0.01(-0.02,0.00)	0.344
Women	0.00 (0.00,0.01)	0.183	0.007 (-0.001,0.02)	0.097
**Gynoid fat (kg)**				
Men	-0.01 (-0.02,0.00)	0.236	- 0.01 (-0.02,0.00)	0.404
Women	0.01 (0.00,0.03)	0.063	0.02 (0.00,0.03)	0.041
**Lean mass percentage (%)**				
Men	0.04 (-0.02,0.10)	0.196	0.05 (-0.02,0.12)	0.164
Women	-0.07 (-0.13,-0.004)	0.038	-0.08 (-0.14,-0.02)	0.006

[a] Analysis adjusted for sex, age, family income, marital status, television and reading time, physical activity level, smoking, anabolic steroid use and total dietary energy intake

TDEI: Total Dietary Energy Intake

Margins plot of ultra-processed foods (UPF) consumption in %grams at 23-25 years (2002-2004) versus Body Mass Index (BMI) (kg/m^2) at 37-39 years (2016-2017) according to sex

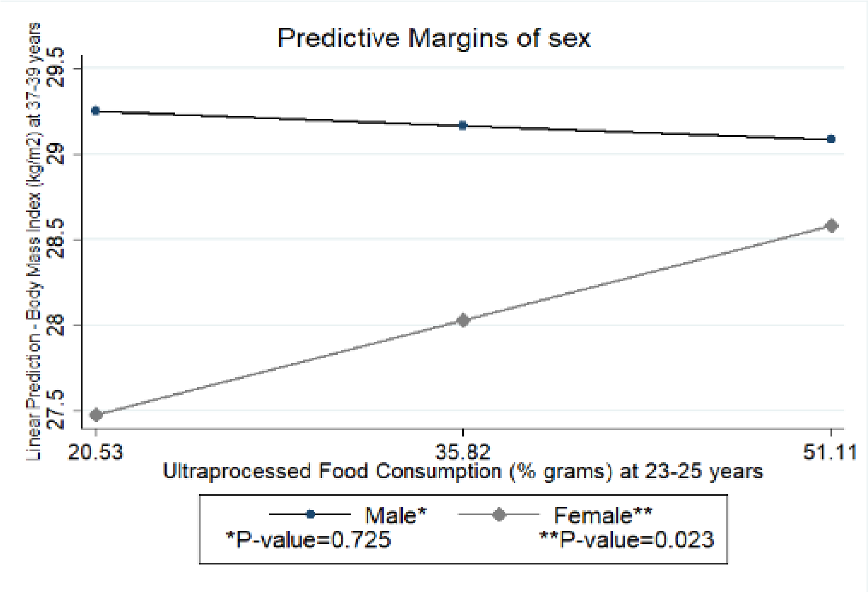

Margins plot of ultra-processed foods (UPF) consumption in %kcal at 23-25 years (2002-2004) versus Body Mass Index (BMI) (kg/m^2) at 37-39 years (2016-2017) according to sex

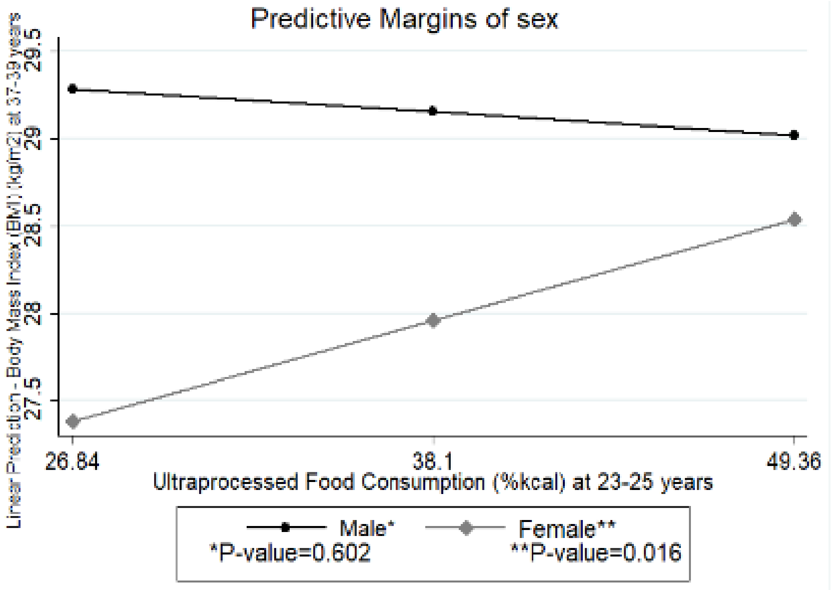

Figure S3. Conditional effects of ultra-processed foods (UPF) consumption at 23-25 years (2002-2004) on body composition measurements - Body Mass Index (kg/m^2) at 37-39 years (2016-2017) according to sex. Ribeirão Preto Cohort 1978/79, São Paulo, Brazil (n=1,021)

Margins plot of ultra-processed foods (UPF) consumption in %grams at 23-25 years (2002-2004) versus Fat Mass Index (kg/m^2) at 37-39 years (2016-2017) according to sex

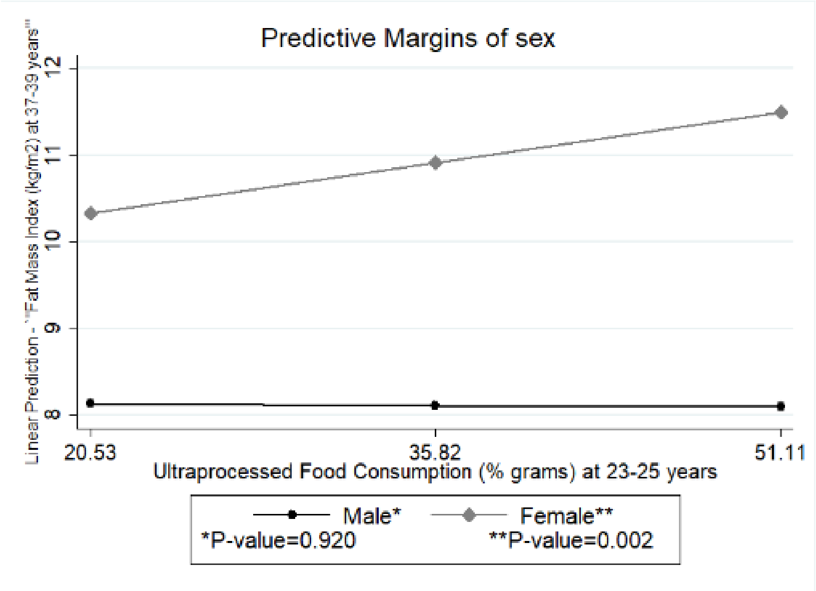

Margins plot of ultra-processed foods (UPF) consumption in %kcal at 23-25 years (2002-2004) versus Fat Mass Index (kg/m^2) at 37-39 years (2016-2017) according to sex

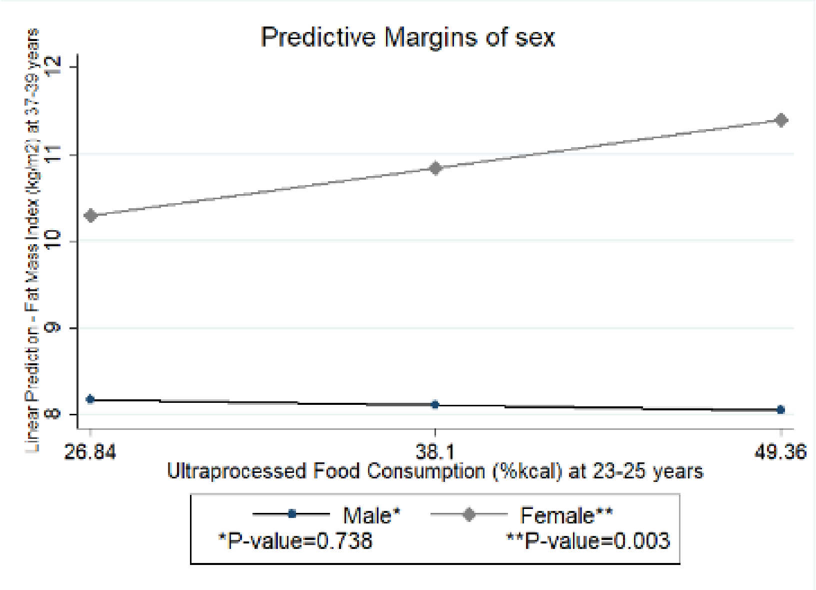

Figure S4. Conditional effects of ultra-processed foods (UPF) consumption at 23-25 years (2002-2004) on body composition measurements - Fat Mass Index (kg/m^2) at 37-39 years (2016-2017) according to sex. Ribeirão Preto Cohort 1978/79, São Paulo, Brazil (n=1,021)

Margins plot of ultra-processed foods (UPF) consumption in %grams at 23-25 years (2002-2004) versus Body Fat (%) at 37-39 years (2016-2017) according to sex

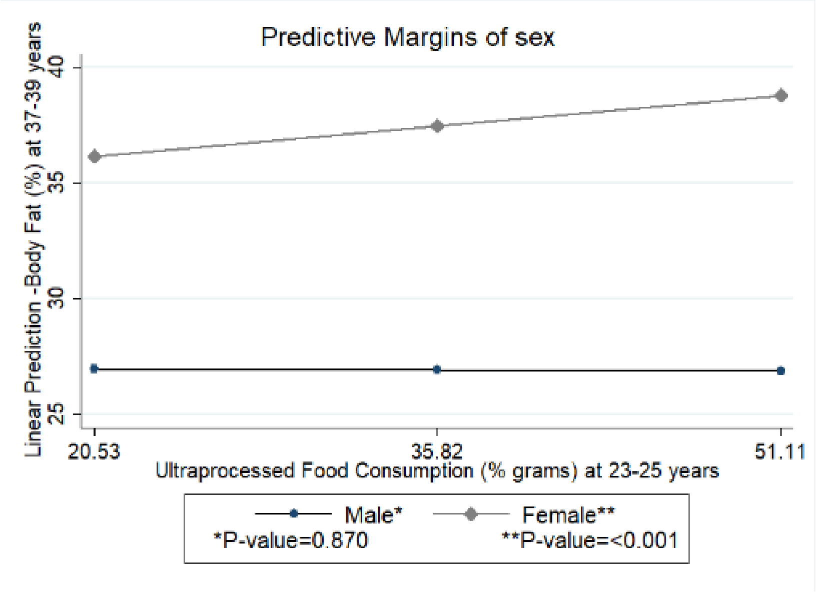

Margins plot of ultra-processed foods (UPF) consumption in %kcal at 23-25 years (2002-2004) versus Body Fat (%) at 37-39 years (2016-2017) according to sex

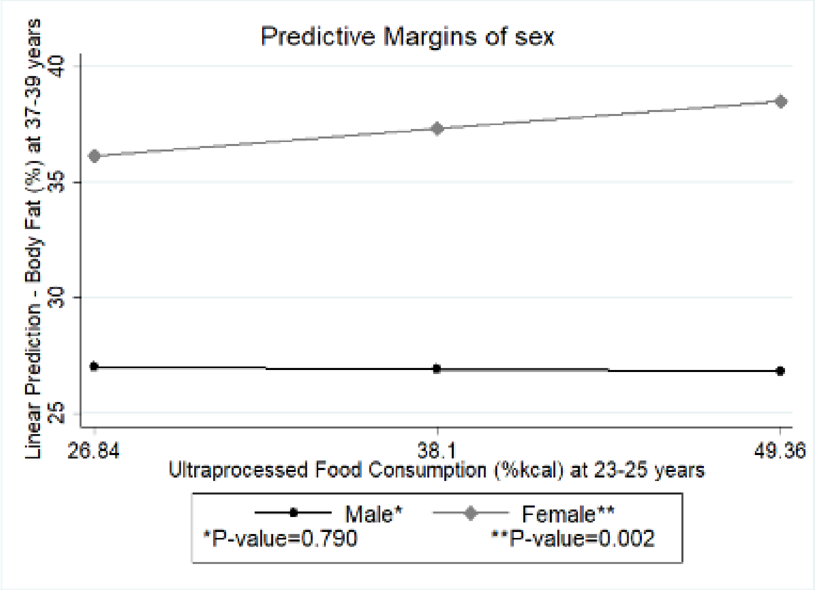

Figure S5. Conditional effects of ultra-processed foods (UPF) consumption at 23-25 years (2002-2004) on body composition measurements - Body Fat (%) at 37-39 years (2016-2017) according to sex. Ribeirão Preto Cohort 1978/79, São Paulo, Brazil (n=1,021)

Margins plot of ultra-processed foods (UPF) consumption in %grams at 23-25 years (2002-2004) versus Android Fat (kg) at 37-39 years (2016-2017) according to sex

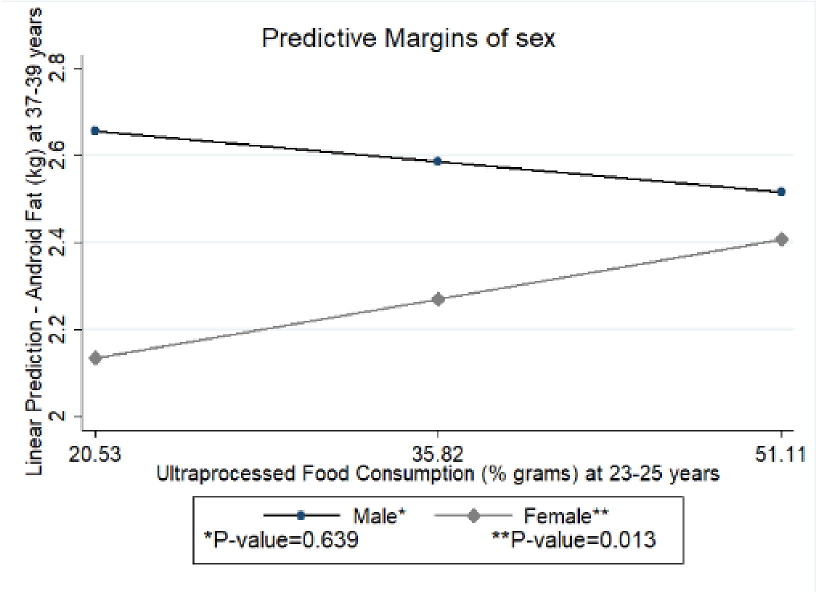

Margins plot of ultra-processed foods (UPF) consumption in %kcal at 23-25 years (2002-2004) versus Android Fat (kg) at 37-39 years (2016-2017) according to sex

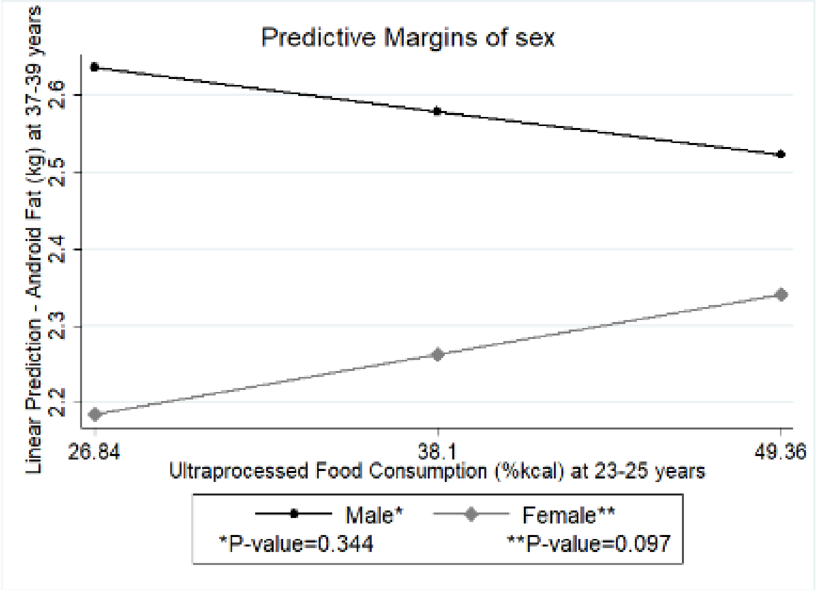

Figure S6. Conditional effects of ultra-processed foods (UPF) consumption at 23-25 years (2002-2004) on body composition measurements – Android Fat (kg) at 37-39 years (2016-2017) according to sex. Ribeirão Preto Cohort 1978/79, São Paulo, Brazil (n=815)

Margins plot of ultra-processed foods (UPF) consumption in %grams at 23-25 years (2002-2004) versus Gynoid Fat (kg) at 37-39 years (2016-2017) according to sex
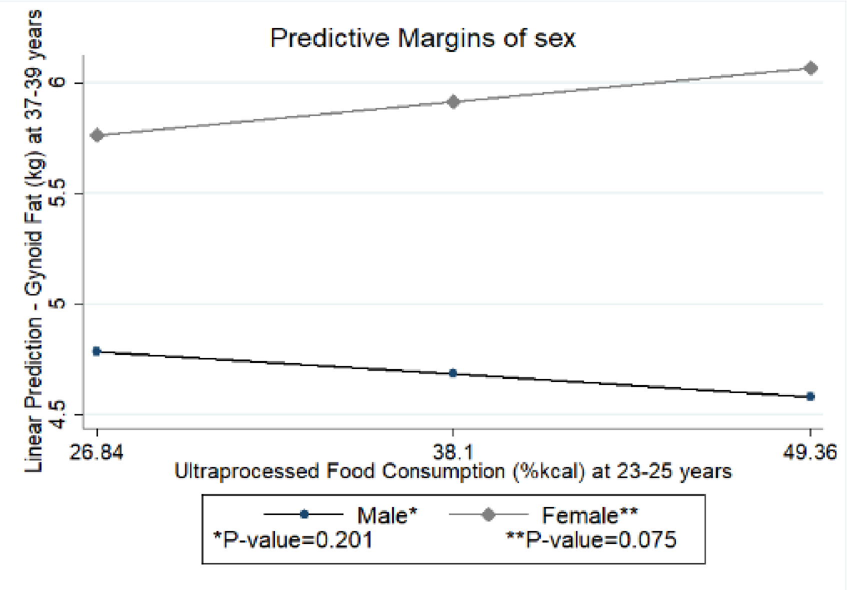
Margins plot of ultra-processed foods (UPF) consumption in %kcal at 23-25 years (2002-2004) versus Gynoid Fat (kg) at 37-39 years (2016-2017) according to sex
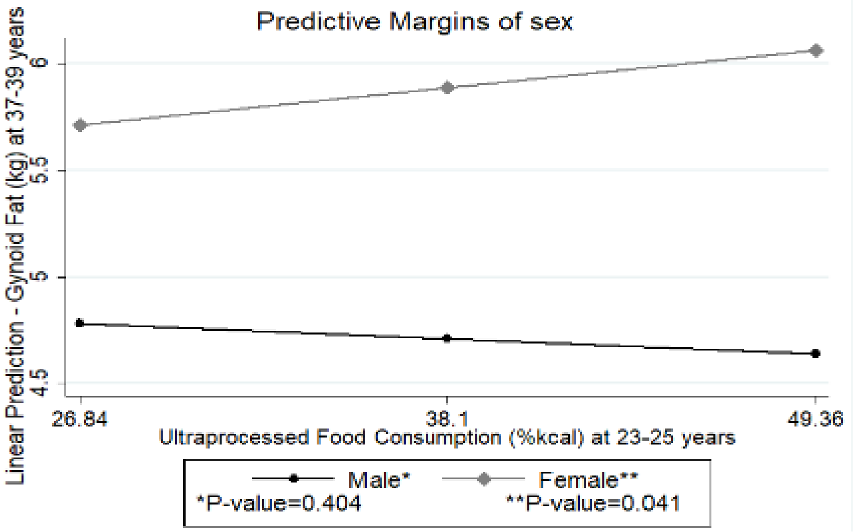

Figure S7. Conditional effects of ultra-processed foods (UPF) consumption at 23-25 years (2002-2004) on body composition measurements –Gynoid Fat (kg) at 37-39 years (2016-2017) according to sex. Ribeirão Preto Cohort 1978/79, São Paulo, Brazil (n=815)

Margins plot of ultra-processed foods (UPF) consumption in %grams at 23-25 years (2002-2004) versus Lean Mass (%) at 37-39 years (2016-2017) according to sex
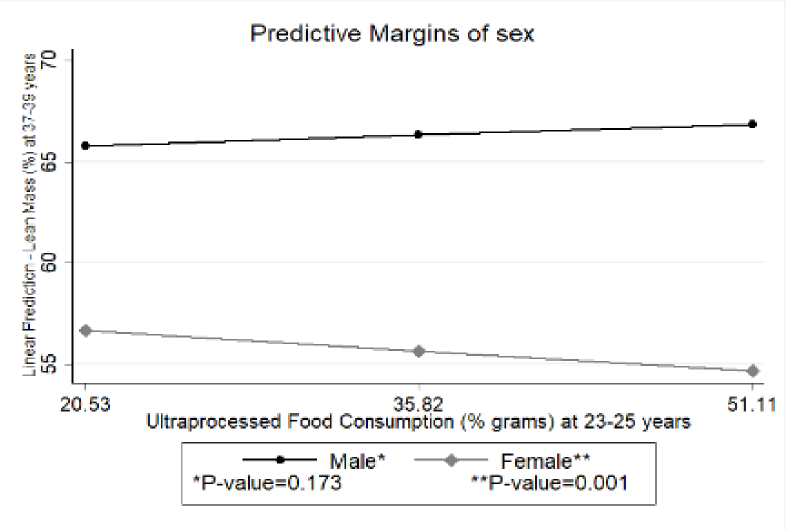
Margins plot of ultra-processed foods (UPF) consumption in %kcal at 23-25 years (2002-2004) versus Lean Mass (%) at 37-39 years (2016-2017) according to sex
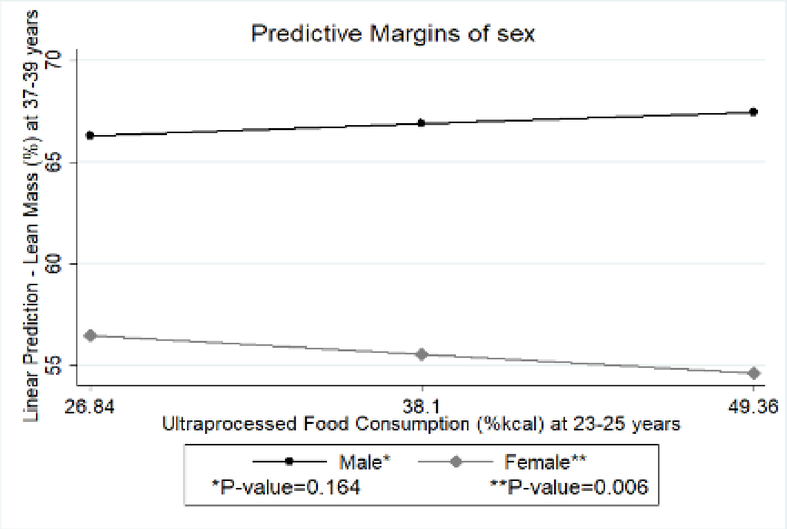

Figure S8. Conditional effects of ultra-processed foods (UPF) consumption at 23-25 years (2002-2004) on body composition measurements – Lean Mass (%) at 37-39 years (2016-2017) according to sex. Ribeirão Preto Cohort 1978/79, São Paulo, Brazil (n=815)
